# Integrated multi-omics reveals distinct maternal and neonatal gut microbial and metabolic signatures associated with small for gestational age status

**DOI:** 10.3389/fmicb.2025.1668148

**Published:** 2025-11-26

**Authors:** Xiangyu Bian, Huisong Xu, Jianqiang Li, Jian Kuang, Fangshu Shi, Xiaoqiong Li, Jinjun Li

**Affiliations:** 1 State Key Laboratory for Quality and Safety of Agro-products & Food Sciences Institute, Zhejiang Academy of Agricultural Sciences, Hangzhou, China; 2 Obstetrical Department of Hangzhou Linping District Maternal & Child Health Care Hospital, Hangzhou, Zhejiang, China

**Keywords:** small for gestational age infants, metagenome, metabolome, tryptophanmetabolism, short-chain fatty acid

## Abstract

**Introduction:**

Children born small for gestational age (SGA) have an elevated risk of developing metabolic disorders in later life. However, the underlying gut microbiota and metabolomic alterations in SGA mother-infant dyads remain poorly understood.

**Methods:**

We performed an integrated analysis of fecal metagenomics, metabolomics, and short-chain fatty acids (SCFAs) profiling in 10 SGA and 10 appropriate for gestational age (AGA) mother-infant dyads at term. Taxonomic composition, microbial functional pathways, carbohydrate-active enzyme (CAZyme) profiles, differential metabolites, and metabolite pathway enrichment were systematically evaluated.

**Results and discussion:**

SGA neonates exhibited reduced microbial richness (Chao1 index), distinct beta-diversity, and differential abundance of key bacterial species including increased Enterococcus faecalis and Escherichia coli. Functionally, SGA maternal subjects showed divergent profiles in CAZyme genes, with lower abundance of glycoside hydrolase family 13 subfamily 16, glycosyl transferase family 66, and carbohydrate-binding module family 6, and altered structural polysaccharide degradation capacity. Metabolomic profiling revealed significant perturbations in tryptophan metabolism pathways, notably enriched in kynurenine and taurine derivatives in SGA mother and neonates. Notably, SCFA profiles were disrupted, with increased butyrate in SGA mother and reduced propionate and isobutyrate in SGA neonates. Microbe-metabolite correlation networks revealed strong associations between SGA-specific bacterial taxa and fecal metabolites. In conclusion, our analysis identifies distinct features of the early fecal microbiome and metabolome within 48 h of birth in SGA neonates compared with AGA peers, reflecting differences in initial colonization and metabolism that warrant longitudinal follow-up.

## Introduction

Low birth weight has been defined as weight at birth of < 2,500 g (5.5 pounds) by the World Health Organization. Small for gestational age (SGA), newborns whose birth weight below the 10th percentile or 2 standard deviations of the many other babies of the same gestational age (Alkalay et al., 1998; Usher and McLean, 1969). SGA infants have become a significant public health concern due to their higher risk of complications compared to appropriate for gestational age (AGA) infants. United Nations International Children's Emergency Fund (2023) reports that 14.7% of newborns globally in 2020 had low birth weight, and its incidence was 17.0% in Asia. The pathogenesis of SGA infants involves multiple factors, including genetics, placental dysfunction, and maternal nutritional status. SGA neonates face higher risks of both short-term and long-term health complications, such as neonatal distress, feeding intolerance, necrotizing enterocolitis, insulin resistance, and an increased likelihood of metabolic disorders in adulthood. Understanding the underlying mechanisms of SGA development is crucial for early interventions and improving long-term health outcomes.

The gut microbiota plays a crucial role in infant health, and its composition, diversity, and abundance vary among neonates with different physiological conditions. Chen et al. (2022) analyzed the changes in gut microbiota of SGA infants from birth to 6 months and found that certain pathogenic and conditional pathogenic bacteria, such as *Escherichia_Shigella, Ralstonia* and *Clostridium* increased. SGA neonates also exhibit lower microbial diversity and a reduced proportion of beneficial bacteria, including *Klebsiella* and *Enterobacter* (Chang et al., 2022). Dietary changes throughout infancy are consistent with increasing demands for carbohydrate-active enzyme (CAZyme). The complex structures of human milk oligosaccharides (HMOs) necessitate a specific repertoire of glycoside hydrolases (GHs), notably those belonging to the GH13 and GH26 families, which are abundant in *Bacteroidetes* and *Bifidobacterium species* (Shoemaker et al., 2001; Xu et al., 2024). CAZyme profiling in SGA infant feces provides functional insight into gut microbial adaptation to nutritional challenges, potentially revealing enzymatic deficiencies or metabolic adaptations that influence growth and health outcomes. Additionally, changes in microbiome were accompanied by changes in fecal metabolites. SGA neonates exhibit increased levels of hexanoylcarnitine alongside reduced abundances of 3-hydroxybutyrate and 3-iminopropanoate, indicating disruptions in taurine metabolism and ketone body pathways (Wang et al., 2022). The perinatal period is a critical window for the establishment of the infant gut microbiota. During the perinatal period, microorganisms in the maternal gut can share genes with those in the infant gut, and this process begins even before birth and continues for several weeks postpartum (Vatanen et al., 2022). This horizontal gene transfer allows maternal microbes to influence the functional properties of the infant's microbiome, even though the microbial strains themselves are not continuously transmitted. However, there is currently no direct evidence confirming whether differences in initial microbial colonization in SGA infants are associated with insufficient or abnormal transmission of the maternal microbiota.

This study aims to investigate the metagenomic and untargeted metabolomic characteristics of the feces in maternal-infant dyads. In addition, to clarify the role of the gut microbiota and metabolites in the development of SGA, we performed gut microbiota functional prediction and correlation analysis between differential microbiota and fecal metabolites. The findings of this study will provide a theoretical basis for the early prediction and effective intervention of SGA infants.

## Subjects and methods

### Subjects

A case-control study design was employed, with women delivering SGA infants matched 1:1 with those delivering AGA infants. Matching criteria included full-term birth (defined as a gestational age between 37 and 42 weeks), body mass index (BMI, kg/m^2^) within the normal range, maternal age within ±5 years, and vaginal delivery. SGA and AGA neonates and their mother were recruited from the pediatric neonatal ward of Hangzhou City Linping District Maternal and Child Care Hospital between July 2021 and February 2022. A total of 20 eligible pregnant women were enrolled, including 10 mothers of SGA infants (SGA_M group) and 10 mother of AGA infants (AGA_M group). Correspondingly, 20 newborns were included, with 10 SGA infants assigned to the SGA_S group and 10 AGA infants to the AGA_S group. Based on intervention data, measurement robustness was quantified as the sample size required to achieve 80% power at α = 0.05 and effect size = 0.60 (two-tailed), calculated *a priori* using G^*^Power 3.19.7. All infants in this study were exclusively breastfed, thereby standardizing feeding mode and eliminating formula-related confounding factors.

### Inclusion criteria

The following inclusion criteria were used: (a) neonates in the SGA group were required to meet the diagnostic criteria of SGA infant: newborns whose birth weight was less than the 10th percentile of the birth weight for the same gestational age and sex; (b) gestational age was defined as ≥37 and < 42 weeks; (c) newborns without asphyxia, neonatal hypoxic-ischemic encephalopathy, intrauterine infection, severe intracranial hemorrhage, neonatal infectious diseases, chromosomal aneuploidies, and genetic metabolic diseases; and (d) informed consent was obtained from the parent or legal guardian of the infant.

### Exclusion criteria

The following exclusion criteria were used: (a) the pregnant women were exposed to antibiotics and probiotics 2 weeks before sample collection; (b) a self-reported special medication history, smoking, or a history of drug or alcohol abuse; (c) presence of severe neonatal conditions such as birth asphyxia, intrauterine infection, hypoxic-ischemic encephalopathy, or neonatal infectious diseases; (d) congenital malformations or genetic disorders in the mother or infant.

## Methods

### Data collection

Clinical data, including sex, gestational age, birth weight, and mode of delivery were collected. Meconium from neonates and stool samples from pregnant women were collected within 48 h after delivery. The specimens were stored in sterile freezing tubes and stored at −80°C for analysis.

### Targeted metabolites measurement of short chain fatty acids (SCFAs)

Quantitative determination of SCFAs was performed as described previously (Li et al., 2025). Briefly, the stool samples were mixed with 50 μL of 15% phosphoric acid, 100 μL of internal standard (isohexanoic acid) solution (125 μg/mL), and 400 μL of ether. The mixture was homogenized for 1 min and then centrifuged at 12,000 rpm and 4°C for 10 min. The supernatant was used for SCFA determination (Trace 1310 GC coupled to a Thermo ISQ mass spectrometer, Thermo Scientific, USA). Chromatographic separation was performed on an Agilent HP-INNOWAX capillary column (30 m × 0.25 mm × 0.25 μm). The concentrations of SCFAs were quantified via a single-point internal standard method. Peak identification and internal response factors were determined using a calibration cocktail containing acetate, propionate, butyrate, isobutyrate, valerate, isovalerate. Concentrations were quantified via an internal standard method. Normalization was performed using a standard curve from QC samples within each batch to eliminate inter-batch differences.

### Stool metagenomic sequencing and analysis

Genomic DNA from 50 mg stool samples were extracted using the QIAamp DNA Stool Mini Kit (Qiagen, Dusseldorf, Germany), following the manufacturer's instructions. After fragmenting the extracted stool DNA, whole-genome shotgun metagenomic sequencing was used to sequence total metagenomic DNA on the Illumina TruSeq Nano DNA LT Library Prep at Suzhou PANOMIX Biomedical Tech (Suzhou, China), using a paired-end 150 bp shotgun sequencing strategy and an insert size of 400 bp. DNA libraries were constructed using a fragmentation-based approach. The library was multiplexed and sequenced using Illumina Hiseq X-ten platform. Libraries were quantified by Qubit TM 3.0 Fluometer (ThermoFisher).

Raw sequencing reads were quality-checked using FastQC. Low-quality reads, adapter sequences, and host-derived contamination were removed using Trimmomatic. High-quality reads were retained for downstream analysis. Cleaned reads were assembled into contigs using MEGAHIT, and gene prediction was performed using Prodigal. Taxonomic classification was assigned using Kraken2 against a curated microbial reference database, while functional annotation was performed using the KEGG and EggNOG databases. Species and functional gene abundance tables were normalized using the centered log-ratio transformation. Sequencing batch was included as a covariate in the generalized linear model to control for its effects.

Alpha and beta diversity indices were calculated using QIIME2 to compare microbial composition between groups. Differentially abundant taxa and functional pathways were identified using linear discriminant analysis effect size (LEfSe) and DESeq2, with a significance threshold of *p* < 0.05. CAZyme family abundances were quantified using Transcripts Per Kilobase Million (TPM) to account for gene length and sequencing depth. Between-sample normalization was performed using DESeq2′s size factors for metagenomic data, and BH-FDR was applied to control multiple testing.

### Metabolomic profiling of stool samples

Chemical compounds in maternal stool samples were assessed by untargeted liquid chromatography-mass spectrometry (LC-MS) at Suzhou PANOMIX Biomedical Tech (Suzhou, China). 50 mg stool sample was added to 600 μL L-2-Chlorophenylalanine in methanol and centrifuged at 12,000 rpm at 4°C for 10 min. The supernatant was filtered through a 0.22 μm membrane. Chromatography was carried out with an ACQUITY UPLC^®^ HSS T3 (150 × 2.1 mm, 1.8 μm) (Waters, Milford, MA, USA), with the column maintained at 40°C. The flow rate and injection volume were set at 0.3 mL/min and 2 μL, respectively. The mobile phases for the gradient conditions were performed as described by Eva et al. (Zelena et al., 2009). Mass spectrometric detection of metabolites was performed on Orbitrap Exploris 120 (Thermo Fisher Scientific, USA). Electrospray ionization mass spectrometric (ESI-MS) experiments were performed with spray voltages of 3.5 and −2.5 kV in positive and negative modes. Sheath and auxiliary gas and capillary temperature were set at 30 and 10 arbitrary units and 325°C. The scanning quality range of the analyzer was m/z 81–1,000, and the mass resolution is 60,000. Raw LC-MS data were converted to mzXML format and processed using XCMS for peak detection, retention time alignment, and normalization. Noise filtering and batch effect correction were applied to improve data reliability. Metabolites were annotated by searching the Human Metabolome (HMDB) Database (www.hmdb.ca).

Raw peak area data were log-transformed. Instrumental drift was corrected based on a regression model established from QC samples, and the ComBat algorithm was employed to adjust for batch effects arising from sample preparation. Multivariate statistical analyses, including principal component analysis (PCA) and orthogonal partial least squares-discriminant analysis (OPLS-DA), were conducted to identify metabolic differences between groups. Differential metabolites were selected based on a variable importance in projection (VIP) score >1.0 and *p* < 0.05. Pathway enrichment analysis was performed using MetaboAnalyst to identify key metabolic pathways associated with the observed changes.

### Ethical approval

The study was approved by the Ethics Committee of the Obstetrical Department of Hangzhou Linping District Maternal &Child Health Care Hospital. The legal guardians of each participant provided written informed consent (Ethics ID: LLSC-KYKT-2022-0048-A).

### Statistical analyses

Demographic characteristics were calculated and presented as median (upper or lower quartile) for continuous variable and proportions for categorical variables. Categorical variables were compared by means of chi-square test. Continuous variables were compared using Students' *t*-test. All analyses were performed with IBM SPSS Statistics (version 25.0) and STATA (version 16.0). Two-tailed *p*-values of < 0.05 were considered statistically significant.

## Results

### Demographics and clinical characteristics of the participants

A total of 10 SGA neonates were enrolled in the SGA_S group, including 7 males. The AGA_S group included 10 AGA neonates, with 6 males. All neonates were born at a gestational age of 37–42 weeks and were fed a diet of breast milk. The clinical characteristics of the enrolled participants are presented in Table 1. Compared to the AGA_S group, neonates in the SGA_S group had significantly higher gestational age and lower birth weight. However, no significant differences were found between the SGA_M and AGA_M groups in terms of maternal BMI, geographic region, maternal education level, presence of siblings. Furthermore, none of the enrolled neonates were infected with the novel coronavirus, and their mother and family members did not exhibit any pandemic-related psychological or behavioral abnormalities.

**Table 1:** Demographics and clinical characteristics of the participants.

	**AGA group**	**SGA group**	***p*-value**
Male	6 (30%)	7 (35%)	>0.99
Birth weight of infants (kg)	3.02 ± 0.25	2.65 ± 0.16	< 0.01
Mother's BMI (kg/m^2^)	21.47 ± 2.95	20.69 ± 3.15	0.57
Gestational age (weeks)	38.80 ± 0.59	39.43 ± 0.62	0.03
Maternal smoking	0 (0%)	0 (0%)	>0.99
Mother's age (years)	30.60 ± 3.41	29.30 ± 5.48	0.53
Prenatal steroid use	0 (0%)	0 (0%)	>0.99
Prenatal antibiotics use	0 (0%)	0 (0%)	>0.99

The *p*-value was calculated using the two-tailed unpaired Students' *t*-test. The *p*-value interaction between groups was corrected by Bonferroni-correction.

### Compositional profiles of the fecal microbiome and taxonomic differences between the AGA and SGA participants

**Figure 1:**
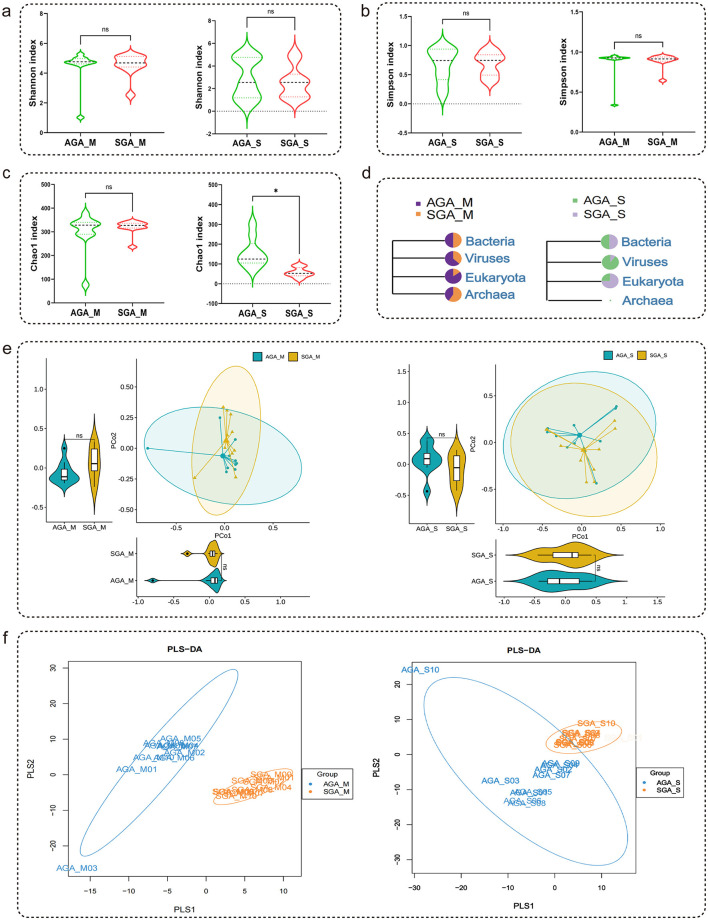
Alpha and beta diversity analysis of microbial communities across different groups. **(a)** Changes in the Shannon index. **(b)** Changes in the Simpson index. **(c)** Changes in the Chao1 index. **(d)** Alternations of microbial domains between AGA and SGA subjects. **(e)** PCoA plot of gut microbiota of subjects according to the Bray-Curtis distance. **(f)** PLS-DA models discriminated microbiome between the AGA and SGA groups. The *p*-value was calculated using the two-tailed unpaired Students' *t*-test. *p < 0.05. The *p*-value interaction between groups was corrected by Bonferroni-correction.

To assess the diversity of gut microbiota in maternal and newborn subjects, the alpha diversity indices (Shannon, Simpson, and Chao1 index) were calculated. There are no significant differences in the Shannon and Simpson indices between AGA_M and SGA_M groups, as well as AGA_S and SGA_S groups (Figures 1a, b). As shown in Figure 1c, Chao1 index was significantly reduced in SGA_S group as compared to the AGA_S group; there was no significant difference between AGA_M and SGA_M groups. The distribution of maternal and neonatal microorganisms included bacteria, viruses, archaea, and eukaryotes (Figure 1d). Principal Coordinates Analysis (PCoA) by bray-curtis distance showed no significant difference between the bacterial profiles of AGA_M and SGA_M groups, as well as AGA_S and SGA_S groups (Figure 1e). Partial least squares discriminant analysis (PLS-DA) revealed distinct differences between AGA_M and SGA_M groups, as well as AGA_S and SGA_S groups, at the bacterial species level (Figure 1f). Therefore, only bacterial taxa were considered for downstream comparative analysis between the AGA and SGA groups in maternal and infant.

**Figure 2:**
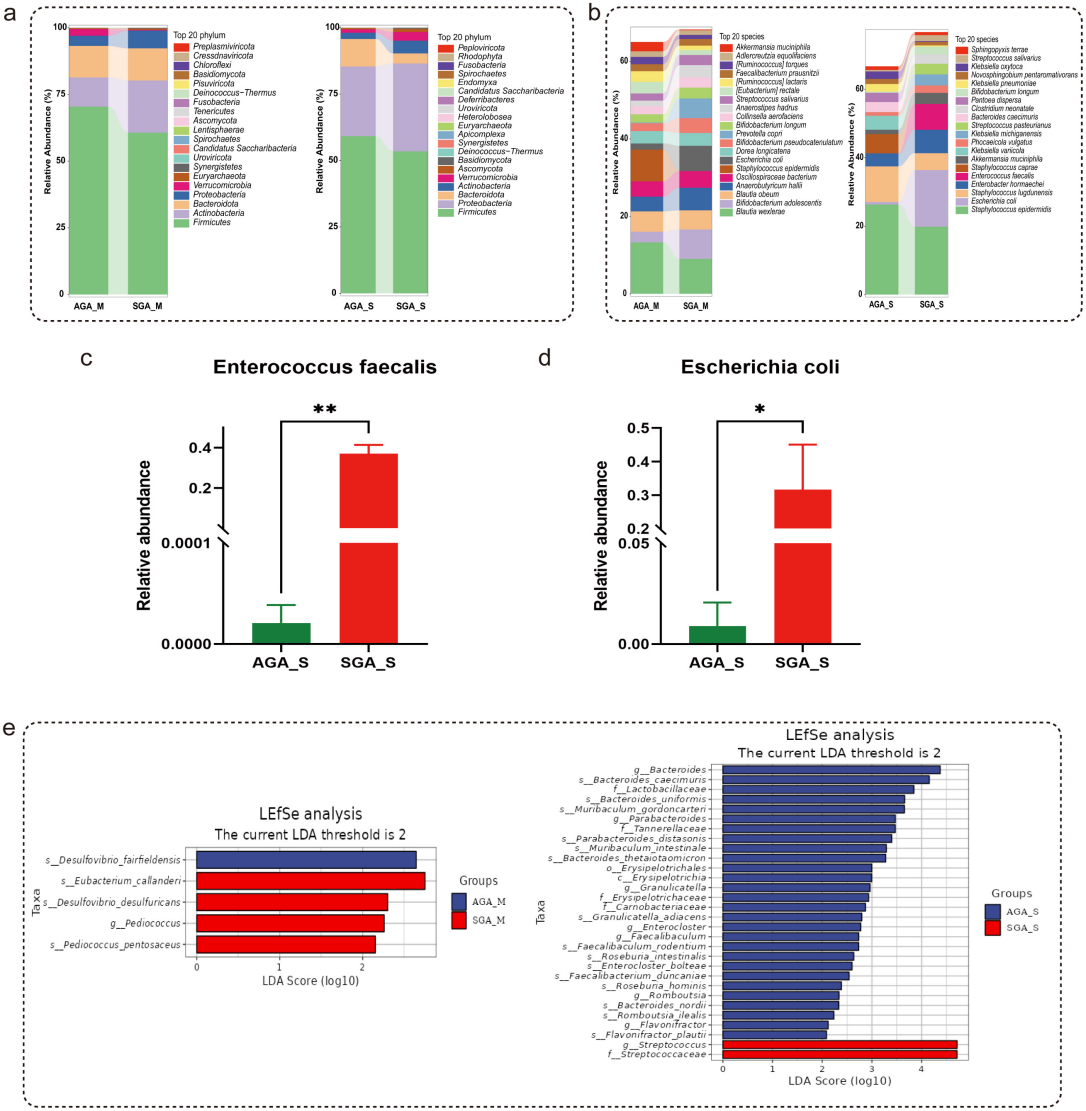
Changes in the taxonomic distribution of AGA and SGA subjects. **(a)** Composition of the microbial community at the phylum. **(b)** Composition of the microbial community at the species level. **(c)** Relative abundance of *Enterococcus faecalis*. **(d)** Relative abundance of *Escherichia coli*. **(e)** LDA score plot based on LEfSe analysis. The *p*-value was calculated using the Kruskal-Walli's test. *p < 0.05. The *p*-value interaction between groups was corrected by Bonferroni-correction. **p < 0.01

At the phylum level, the relative abundance of Tenericutes increased significantly in SGA_M group when compared to AGA_M group (*p* < 0.05, by a Wilcoxon rank sum test, Figure 2a, left). Compared to the AGA_S group, the relative abundance of Synergistetes was significantly decreased in the SGA_S group (*p* < 0.05, by a Wilcoxon rank sum test, Figure 2a, right). At the species level, compared to the AGA_S group, the relative abundance of *Desulfovibrio fairfieldensis, Actinomyces sp.HMT 175, Candidatus Saccharibacteria bacterium*, and *Bacteroides zhangwenhongii* were significantly increased, while *Pediococcus pentosaceus, Uncultured Tenericutes bacterium, Bifidobacterium angulatum, Prevotella jejuni, Streptococcus sp. NCTC 11567, Citrobacter freundii*, and *Desulfovibrio desulfuricans* were significantly decreased in the SGA_M (*p* < 0.05, by a Wilcoxon rank sum test, Table 2). Compared to the AGA_S group, the relative abundance of *Bacteroides caecimuris, Muribaculaceae bacterium MF13079, Firmicutes bacterium ASF500, Muribaculum gordoncarteri, Duncaniella dubosii, Faecalibacterium duncaniae, Bacillus pacificus, Roseburia intestinalis, Bacteroides nordii, Romboutsia ilealis, Flavonifractor plautii, Roseburia hominis, Parabacteroides goldsteinii*, and *Granulicatella adiacens* were significantly increased, while *Clostridioides difficile, Acinetobacter* sp. *MYb10, Clostridium perfringens, Acinetobacter johnsonii, Streptococcus ilei*, and *Bifidobacterium breve* were significantly decreased in the SGA_S (*p* < 0.05, by a Wilcoxon rank sum test, Table 3). Additionally, the relative abundance of *Enterococcus faecalis* and *Escherichia coli* increased significantly in SGA_S group when compared to AGA_S group (*p* < 0.05, by a Students' *t*-test, Figures 2c, d).

**Table 2:** Significantly differential microbes at species level in the metagenomic sequencing datasets between the AGA_M and SGA_M group.

**Taxon**	**logFC**	***p* values**	**Adjust *p* values**	**Gram stain**	**Oxygen tolerance**	**Metabolite production**
*Desulfovibrio fairfieldensis*	−3.478	0.001624	0.5079	Negative	Anaerobe	Unknown
*Actinomyces sp.HMT 175*	−2.234	0.01168	0.9977	Positive	Unknown	Unknown
*Candidatus Saccharibacteria bacterium*	−2.091	0.02454	0.9977	Unknown	Unknown	Unknown
*Bacteroides zhangwenhongii*	−1.729	0.02995	0.9977	Unknown	Unknown	Unknown
*Pediococcus pentosaceus*	1.822	0.04064	0.9977	Positive	Anaerobe	Unknown
*Uncultured Tenericutes bacterium*	1.903	0.02499	0.9977	Unknown	Unknown	Unknown
*Bifidobacterium angulatum*	1.925	0.01438	0.9977	Positive	Anaerobe	Unknown
*Prevotella jejuni*	2.17	0.02077	0.9977	Negative	Anaerobe	Indole
*Streptococcus sp. NCTC 11567*	2.885	0.009154	0.9977	Positive	Facultative anaerobe	Acetoin
*Citrobacter freundii*	3.074	0.003228	0.5079	Negative	Aerobe	Unknown
*Desulfovibrio desulfuricans*	3.114	0.003162	0.5079	Negative	Anaerobe	Unknown

The *p*-value was calculated by Wilcoxon rank-sum test. Significant *p*-values were corrected using the Benjamini–Hochberg method.

**Table 3:** Significantly differential microbes at species level in the metagenomic sequencing datasets between the AGA_S and SGA_S group.

**Taxon**	**logFC**	***p*-values**	**Adjust *p*-values**	**Gram stain**	**Oxygen tolerance**	**Metabolite production**
*Bacteroides caecimuris*	−5.564	4.69E-05	0.0740	Negative	Anaerobe	Indole
*Muribaculaceae bacterium MF13079*	−4.238	0.005835	0.6088	Unknown	Unknown	Unknown
*Firmicutes bacterium ASF500*	−3.831	0.003544	0.4437	Unknown	Unknown	Unknown
*Muribaculum gordoncarteri*	−3.298	0.04251	0.6801	Negative	Anaerobe	Unknown
*Duncaniella dubosii*	−3.191	0.01279	0.6801	Negative	Anaerobe	Unknown
*Faecalibacterium duncaniae*	−2.931	0.01881	0.6801	Positive	Anaerobe	Indole
*Bacillus pacificus*	−2.871	0.04813	0.6801	Positive	Facultative anaerobe	Acetoin, hydrogen sulfide, indole
*Roseburia intestinalis*	−2.555	0.03158	0.6801	Positive	Anaerobe	Indole
*Bacteroides nordii*	−2.38	0.01623	0.6801	Negative	Anaerobe	Indole
*Romboutsia ilealis*	−2.365	0.01837	0.6801	Positive	Anaerobe	Unknown
*Flavonifractor plautii*	−2.25	0.009774	0.6801	Variable	Anaerobe	Acetoin, indole
*Roseburia hominis*	−2.147	0.04008	0.6801	Positive	Anaerobe	Unknown
*Parabacteroides goldsteinii*	−1.911	0.02461	0.6801	Negative	Anaerobe	Indole
*Granulicatella adiacens*	−1.756	0.0478	0.6801	Positive	Anaerobe	Indole
*Clostridioides difficile*	2.183	0.04078	0.6801	Positive	Anaerobe	Indole
*Acinetobacter sp.MYb10*	2.214	0.03894	0.6801	Unknown	Unknown	Unknown
*Clostridium perfringens*	2.503	0.04459	0.6801	Positive	Anaerobe	Indole
*Acinetobacter johnsonii*	2.727	0.02132	0.6801	Negative	Aerobe	Indole
*Streptococcus ilei*	3.387	0.02597	0.6801	Unknown	Unknown	Unknown
*Escherichia coli*	3.697	0.0003274	0.0127	Negative	Facultative anaerobe	Indole, acetoin, hydrogen sulfide, nitrite
*Bifidobacterium breve*	6.123	0.001969	0.3082	Positive	Anaerobe	Indole
*Enterococcus faecalis*	7.613	0.0002324	0.0014	Positive	Microaerophile	Acetoin, indole

The *p*-value was calculated by Wilcoxon rank-sum test. Significant *p*-values were corrected using the Benjamini–Hochberg method.

The LEfSe algorithm was used to assess differences in microbial abundance between AGA_M and SGA_M groups, as well as AGA_S and SGA_S groups (Figure 2e). The differential species score chart revealed that *Desulfovibrio fairfieldensis* was relatively abundant in the AGA_M group, whereas *Eubacterium callanderi* was more abundant in the SGA_M group. In the AGA_S group, the genus *Bacteroides* and *Bacteroides caecimuris* were enriched, while the genus *Streptococcus* was more prevalent in the SGA_S group (LDA score > 2). Take together, these findings suggest altered microbial ecosystems associated with SGA.

### Microbial function differences between the AGA and SGA participants

**Figure 3:**
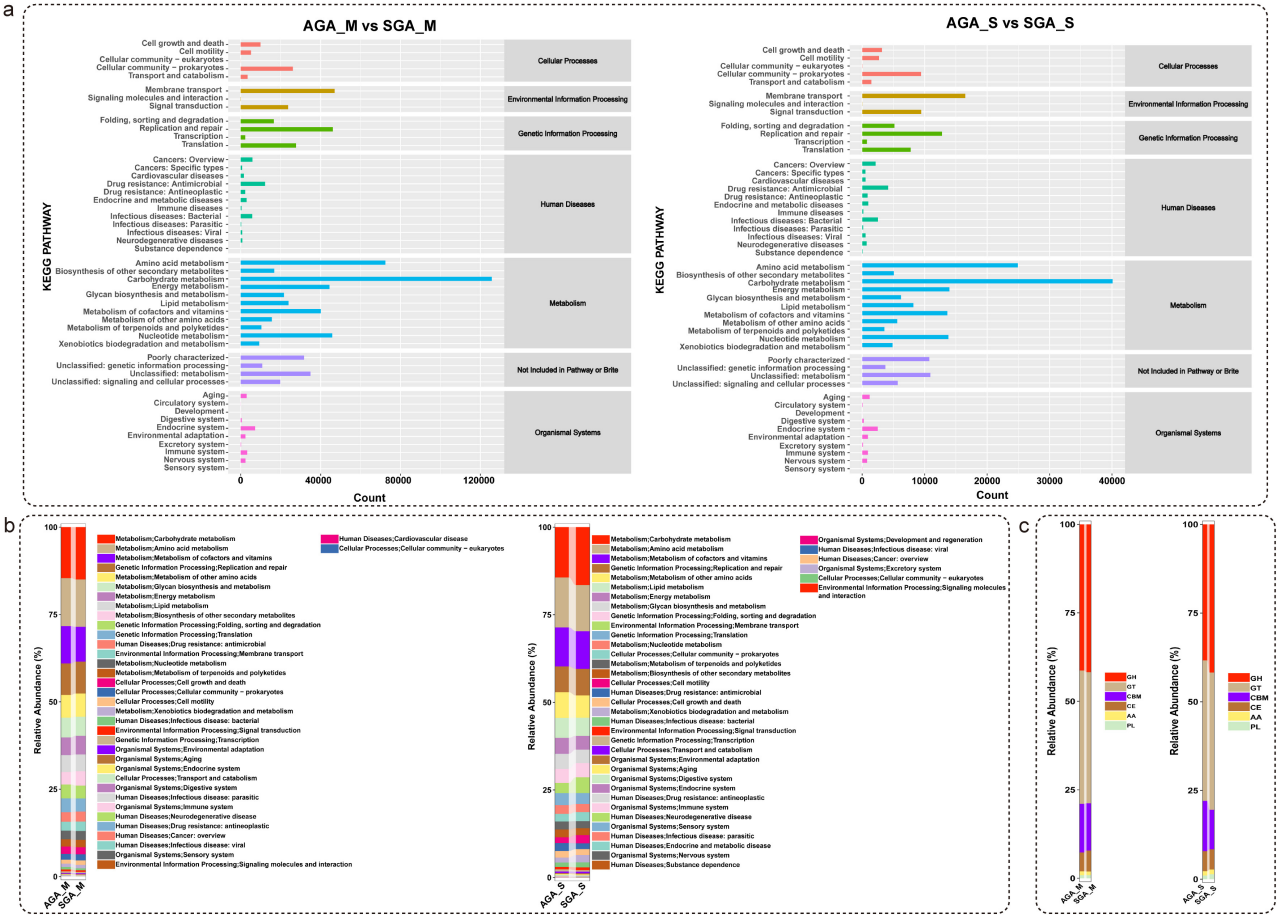
Functional annotation and KEGG pathway enrichment analysis. **(a)** KEGG classification of annotated genes across samples, showing predominant enrichment in metabolic pathways, particularly amino acid metabolism, carbohydrate metabolism, and energy metabolism; **(b)** Relative abundance of functional categories based on KEGG annotations across different groups, with metabolism-related pathways dominating; **(c)** Proportions of major KEGG categories in each group, highlighting metabolism as the primary functional category.

Functional annotation and KEGG pathway enrichment analyses were performed to characterize the biological functions of the annotated genes. As shown in Figure 3a, most genes were primarily associated with metabolism-related pathways, including amino acid metabolism, carbohydrate metabolism, and energy metabolism. Other enriched categories included cellular processes, environmental information processing, and human diseases, although to a lesser extent. Figure 3b further illustrates the relative abundance of functional categories across different groups. Metabolic pathways were dominant in all groups, with notable contributions from carbohydrate metabolism, amino acid metabolism, and lipid metabolism.

To further investigate the microbial potential for carbohydrate degradation in the AGA and SGA subjects, we screened the assembled contigs for the presence of CAZymes. The distribution of different CAZyme classes, including GH, glycosyl transferases (GT), carbohydrate-binding modules (CBM), carbohydrate esterases (CE), auxiliary activities (AA), and polysaccharide lyases (PL), is presented in Figure 3c. The most abundant enzyme class in was the GHs followed by GTs. Less abundant were CBMs, CEs, enzymes with AAs, and PLs.

GHs are responsible for the degradation or hydrolysis of glycosidic bonds in carbohydrates. Oligosaccharide-degrading enzymes are major parts of GH families. Compared with the AGA_M group, we found that the abundance of GH15_2 was significantly increased (*p* < 0.01), and GH20 and GH13_16 were significantly decreased in the SGA_M group (Table 4, *p* < 0.05). Debranching enzymes were identified belonging to GH5_9, GH95, GH163, GH166, GH117, and GH110 families. GH5_9 and GH163 were more abundant (*p* < 0.05) in the SGA_S group than in the AGA_S group, and the abundance of GH95, GH166, GH117, and GH110 in the SGA_S group were lower than in the AGA_S group (Table 5, *p* < 0.05).

**Table 4:** Significantly differential genes encoding GHs between the AGA_M and SGA_M group.

**CAZy family**	**Major activity**	**log_2_ (FC)**	***p*-values**	**FDR**
GH20	b-N-acetylhexosaminidase	−0.274	0.0079	0.8804
GH13_16	Trehalose synthase/maltose glucosylmutase	−2.439	0.0444	0.8804
GH15_2	Cellulose endo-b-1,4-glucosidase	0.888	0.0480	0.8804
GT66	Oligosaccharyltransferase	−0.446	0.0478	0.8804
CBM77	Pectin binding	1.706	0.0463	0.8804

The *p*-value was calculated using the two-tailed unpaired Students' *t*-test. The *p*-value interaction between groups was corrected by Bonferroni-correction.

**Table 5:** Significantly differential genes encoding GHs between the AGA_S and SGA_S group.

**CAZy family**	**Major activity**	**log_2_ (FC)**	***p*-values**	**FDR**
GH5_9	Endo-β-1,4-mannanase	0.775	0.0149	0.6703
GH95	a-L-fucosidase	−2.766	0.0271	0.6703
GH63	a-glucosidase	2.179	0.0273	0.6703
GH117	b-galactofuranosidase	−3.216	0.0362	0.6703
GH66	Dextran endo-a-1,6-glucosidase	−2.945	0.0361	0.6703
GH110	Antigen a-1,3-galactosidase	−6.148	0.0496	0.6703
GT5	Glycogen synthase	−0.740	0.0229	0.6703
GT100	Gal-β-1,3-GalNAc-α-PP-lipid α-2,3-sialyltransferase	−6.423	0.0376	0.6703
GT33	Chitobiosyldiphosphodolichol β-mannosyltransferase	−0.971	0.0479	0.6703
PL1	Pectate lyase	2.931	0.0489	0.6703
CBM6	Cellulose and β-1,4-xylan binding	−0.786	0.0262	0.6703

The *p*-value was calculated using the two-tailed unpaired Students' *t*-test. The *p*-value interaction between groups was corrected by Bonferroni-correction.

PLs employ unique mechanisms to degrade polysaccharides containing uronic acids. Furthermore, the abundance of PL1 in the SGA_S group were higher than in the AGA_S group (Table 5, *p* < 0.05).

GTs are responsible for the addition of saccharides onto other biomolecules. The abundance of GT66 in the SGA_M group was lower than in the AGA_M group (Table 4, *p* < 0.05). Compared with the AGA_S group, we found that the abundance of GT75, GT100, and GT33 genes were significantly decreased in SGA_S group (Table 5).

The CBM domain facilitates the binding of CAZymes to carbohydrate substrates, thereby enhancing enzymatic activity. Compared with the AGA_M group, we found that the abundance of CBM77 gene was significantly increased in SGA_M group (Table 4). The abundance of CBM6 in the SGA_S group was lower than in the AGA_S group (Table 5, *p* < 0.05). These data indicate divergent microbial capacities for carbohydrate metabolism and structural polysaccharide degradation in the SGA subjects.

### Metabolomic variations in AGA and SGA neonatal meconium and maternal feces

**Figure 4:**
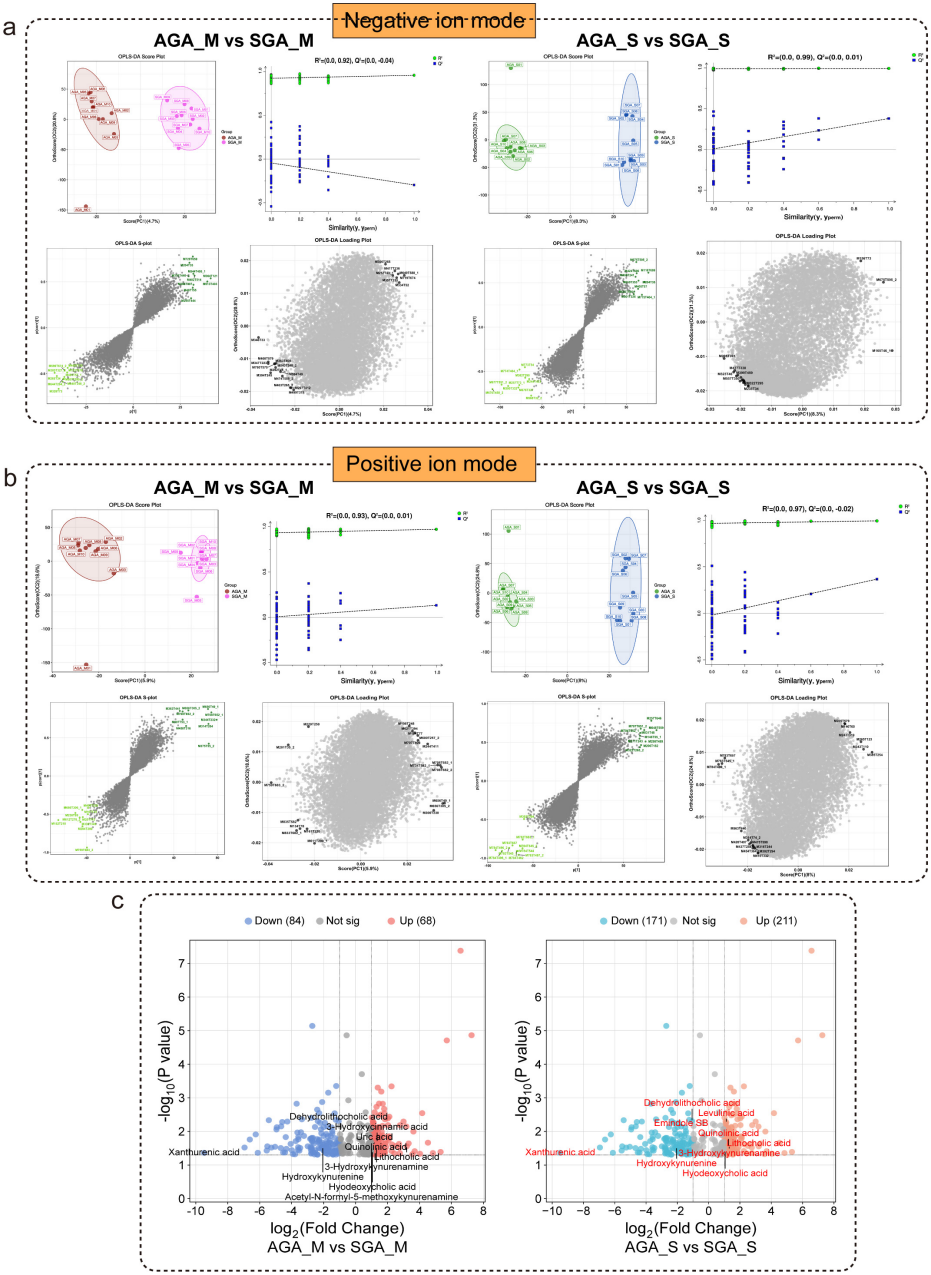
Changes in the fecal metabolome of AGA and SGA subjects. OPLS-DA score plots, permutation test, S-plots, and loading plots comparing metabolic profiles between AGA and SGA groups under negative ion mode **(a)** and positive ion mode **(b)**. Symbols representing samples from the two groups are shown in different colors. **(c)** Significant differential metabolites between the AGA and SGA groups in maternal **(left)** and newborn **(right)** subjects, detected by LC-MS. The x-axis shows fold change (log2 ratio scale), and the y-axis shows the negative log10 of *p*-values (a higher value represents a stronger statistical significance). Significant *p*-values were corrected using the Benjamini–Hochberg method.

To characterize metabolic alterations associated with SGA status, orthogonal partial least squares discriminant analysis (OPLS-DA) was performed in both negative and positive ion modes. Clear group separation was observed between SGA and AGA subjects (Figures 4a, b), indicating distinct metabolic signatures. The OPLS-DA score plots revealed robust model performance between AGA_M and SGA_M groups (*R*^2^ = 0.92 and 0.93 for negative and positive ion modes, respectively), while the neonatal samples (AGA_S vs SGA_S) showed moderate separation (*R*^2^ = 0.99 and 0.97, respectively), although with relatively low predictive power (*Q*^2^ values < 0.05). Although OPLS-DA revealed group separation, the low *Q*^2^ value suggests limited predictive performance and a risk of overfitting.

Volcano plot analysis identified multiple significantly dysregulated metabolites between AGA and SGA participants (Figure 4c). One hundred and fifty two differential metabolites were detected between AGA_M and SGA_M groups. A total of 382 differential metabolites were identified between AGA_S and SGA_S groups. Compared to AGA_M group, quantification levels of 3-hydroxykynurenamine, xanthurenic acid, citrulline, and oxoglutaric acid were significantly increased, while acetyl-N-formyl-5-methoxykynurenamine was decreased in SGA_M group (*p* < 0.05, Table 6). Whereas, in infants, SGA_S subjects exhibited significantly higher levels of xanthurenic acid, indole-3-lactic acid, and citraconic acid and lower level of acetyl-N-formyl-5-methoxykynurenamine and 3-hydroxykynurenamine relative to AGA_S counterparts (*p* < 0.05, Table 7), Collectively, these results demonstrate that metabolic profiles are significantly altered in SGA maternal and neonatal samples, reflecting systemic metabolic perturbations potentially linked to fetal growth restriction.

**Table 6:** Significantly differential metabolites between the AGA_M and SGA_M group.

**Name**	**Formula**	**AGA_M Mean**	**SGA_M Mean**	**FC**	**log_2_FC**	***p*-value**	**FDR**	**VIP**
Xanthurenic acid	C10H7NO4	6,857.39	50,199.62	0.14	−2.87	0.0208	0.77	2.27
L-Ornithuric acid	C19H20N2O4	1,502,090.57	5,587,998.59	0.27	−1.90	0.0142	0.67	2.06
3-Methoxytyrosine	C9H12N2O2	3,651,208.27	12,628,120.37	0.29	−1.80	0.0305	0.86	2.14
3-Hydroxykynurenamine	C10H13NO4	3,731,903.76	13,004,876.30	0.29	−1.79	0.0095	0.59	2.23
Ursolic acid	C10H18N2O5	19,511,745.72	58,705,971.21	0.33	−1.58	0.0010	0.20	2.88
gamma-Glutamylvaline	C30H48O3	2,289,030.74	6,865,880.20	0.33	−1.59	0.0383	0.89	2.18
3a,7b,21-Trihydroxy-5b-cholanoic acid	C24H40O6	15,607,568.45	47,749,072.79	0.33	−1.61	0.0012	0.22	2.83
4-Guanidinobutanoic acid	C5H11N3O2	3,763,318.20	10,228,034.59	0.37	−1.44	0.0275	0.82	2.05
3-Hydroxycinnamic acid	C9H8O3	246,681,122.02	530,936,482.72	0.46	−1.11	0.0243	0.80	2.21
4-Hydroxyretinoic acid	C20H28O3	5,097,147.32	10,826,601.63	0.47	−1.09	0.0327	0.86	2.23
cis,cis-3-Carboxymuconic acid	C7H6O6	11,885,369.25	22,278,092.09	0.53	−0.91	0.0451	1.00	2.11
Urocanic acid	C6H6N2O2	19,714,709.29	36,230,324.70	0.54	−0.88	0.0341	0.87	2.04
5-Hexyl-3,4-dimethyl-2-furannonanoic acid	C21H36O3	3,703,659.46	6,007,735.68	0.62	−0.70	0.0236	0.79	2.15
Citrulline	C6H13N3O3	232,939,004.43	360,155,740.06	0.65	−0.63	0.0468	0.91	1.83
Oxoglutaric acid	C5H6O5	3,813,934.43	4,854,110.79	0.79	−0.35	0.0243	1.00	2.43
Nogalonic acid	C20H14O8	646,851.03	788,123.54	0.82	−0.28	0.0300	1.00	2.48
Sulfoacetic acid	C2H4O5S	7,343,473.94	8,325,020.72	0.88	−0.18	0.0441	1.00	2.37
Ribonic acid	C5H10O6	21,839,682.40	23,266,508.56	0.94	−0.09	0.0122	0.63	2.27
10,11-epoxy-3,11-dimethyl-7-ethyl-2,6-tridecadienoic acid	C17H28O3	19,209,146.06	10,180,013.85	1.89	0.92	0.0135	1.00	2.78
1,4,5,6-Tetrahydro-6-oxonicotinic acid	C6H7NO3	256,508,942.08	118,284,426.90	2.17	1.12	0.0366	0.87	1.97
Cysteic acid	C3H7NO5S	2,866,722.95	1,212,756.58	2.36	1.24	0.0200	0.77	2.25
Acetyl-N-formyl-5-methoxykynurenamine	C13H16N2O4	904,641,088.63	289,325,595.90	3.13	1.64	0.0293	1.00	1.92
Mevalonic acid-5P	C6H13O7P	1,050,035.86	24,813.13	42.32	5.40	0.0278	1.00	2.22

The *p*-value was calculated using the two-tailed unpaired Students' *t*-test. The *p*-value interaction between groups was corrected by Bonferroni-correction.

**Table 7:** Significantly differential metabolites between the AGA_S and SGA_S group.

**Name**	**Formula**	**aga_s mean**	**SGA_S mean**	**FC**	**log_2_FC**	***p*-value**	**FDR**	**VIP**
Xanthurenic acid	C10H7NO4	46,895.97	33,417,802.06	0	−9.48	0.0434	0.47	1.64
4-Guanidinobutanoic acid	C5H11N3O2	63,159.88	4,821,102.00	0.01	−6.25	0.0123	0.46	2.07
(R)-mandelic Acid	C8H8O3	678,039.19	8,225,871.83	0.08	−3.60	0.0351	0.44	1.60
Indole-3-lactic acid	C11H11NO3	1,380,600.40	15,863,856.00	0.09	−3.52	0.0375	0.47	1.64
2-Ethyl-2-Hydroxybutyric acid	C6H12O3	3,621,823.64	28,820,570.24	0.13	−2.99	0.0278	0.46	1.87
3-Hydroxyglutaric acid	C5H8O5	1,630,301.88	11,237,096.48	0.15	−2.79	0.0357	0.44	1.60
17-Methyloctadecanoic acid	C19H38O2	3,369,043.62	22,573,934.43	0.15	−2.74	0.0332	0.46	1.71
Tyromycic acid	C30H44O3	1,231,550.51	6,507,611.11	0.19	−2.40	0.0022	0.31	2.32
Argininosuccinic acid	C10H18N4O6	30,729.17	157,369.28	0.2	−2.36	0.0050	0.37	2.14
Pantothenic acid	C9H17NO5	79,333,581.14	365,589,537.64	0.22	−2.20	0.0324	0.46	1.67
alpha-Hydroxyisobutyric acid	C4H8O3	7,961,675.40	34,486,835.91	0.23	−2.11	0.0291	0.44	1.71
Hydroxykynurenine	C10H12N2O4	15,842,427.17	65,740,054.20	0.24	−2.05	0.0295	0.44	1.72
3-Hydroxy-4-methoxyphenyllactic acid	C10H12O5	214,130.47	854,387.51	0.25	−2.00	0.0136	0.46	1.87
1-Pyrroline-5-carboxylic acid	C5H7NO2	4,781,041.75	18,817,568.10	0.25	−1.98	0.0384	0.47	1.69
Emindole SB	C28H39NO	1,229,902.39	4,173,721.04	0.29	−1.76	0.0072	0.44	2.03
8-Amino-7-oxononanoic acid	C9H17NO3	2,106,181.41	6,901,328.76	0.31	−1.71	0.0481	0.48	1.68
Lovastatin acid	C24H38O6	19,661,191.51	63,992,774.59	0.31	−1.70	0.0368	0.44	1.70
Dehydrolithocholic acid	C24H38O3	34,289,253.50	71,566,009.01	0.48	−1.06	0.0050	0.37	2.16
N-Acetyl-L-glutamic acid	C7H11NO5	9,585,233.84	19,404,126.81	0.49	−1.02	0.0400	0.47	1.63
2-Keto-3-deoxy-6-phosphogluconic acid	C6H11O9P	67,224.46	126,955.22	0.53	−0.92	0.0167	0.44	1.83
Citraconic acid	C5H6O4	16,756,424.86	28,903,288.52	0.58	−0.79	0.0462	0.44	1.52
Phthalic acid	C8H6O4	24,886,752.39	36,665,986.08	0.68	−0.56	0.0000	0.12	2.83
Phosphoroselenoic acid	H3O3PSe	3,036,531.40	4,142,979.33	0.73	−0.45	0.0012	0.23	2.48
L-2-Amino-4-methylenepentanedioic acid	C6H9NO4	7,152,147.69	3,999,649.13	1.79	0.84	0.0454	0.44	1.60
Caffeic acid	C9H8O4	194,820,610.58	107,395,480.61	1.81	0.86	0.0129	0.46	1.94
Quinic acid	C7H12O6	30,930,571.01	15,996,840.95	1.93	0.95	0.0395	0.44	1.66
Acetyl-N-formyl-5-methoxykynurenamine	C13H16N2O4	1,009,338,380.21	506,289,760.49	1.99	1.00	0.0303	0.44	1.70
Palmitic acid	C16H32O2	233,327,216.09	114,457,970.63	2.04	1.03	0.0091	0.43	2.04
Gibberellin A44 diacid	C20H28O6	48,566,354.42	23,425,378.73	2.07	1.05	0.0270	0.44	1.71
Hyodeoxycholic acid	C24H40O4	133,124,328.24	64,105,506.43	2.08	1.05	0.0388	0.44	1.64
Dodecanedioic acid	C12H22O4	298,449,435.45	138,336,197.62	2.16	1.11	0.0130	0.44	2.02
Levulinic acid	C5H8O3	7,501,565.10	3,417,223.16	2.2	1.13	0.0055	0.44	2.05
Threonic acid	C4H8O5	14,121,463.24	6,304,721.21	2.24	1.16	0.0100	0.44	2.17
Uric acid	C5H4N4O3	182,283,401.74	80,281,141.33	2.27	1.18	0.0223	0.44	1.84
Pomonic acid	C30H46O4	10,447,218.92	4,517,713.53	2.31	1.21	0.0242	0.44	1.88
Ganolucidic acid A	C30H44O6	59,5035,134.45	248,100,418.04	2.4	1.26	0.0081	0.44	2.04
Quinolinic acid	C7H5NO4	2,408,343.23	1,000,945.59	2.41	1.27	0.0278	0.44	1.86
Chlorogenic acid	C16H18O9	52,921,903.91	21,342,017.13	2.48	1.31	0.0019	0.40	2.31
3-Hydroxykynurenamine	C9H12N2O2	883,597.89	355,827.18	2.48	1.31	0.0291	0.46	1.84
3-Hydroxycinnamic acid	C9H8O3	766,217,874.19	300,117,512.77	2.55	1.35	0.0206	0.46	1.92
Lithocholic acid	C24H40O3	636,619,095.49	69,181,943.71	9.2	3.20	0.0278	0.46	1.72

The *p*-value was calculated using the two-tailed unpaired Students' *t*-test. The *p*-value interaction between groups was corrected by Bonferroni-correction.

### Altered metabolic pathway enrichment distinguishes SGA and AGA subjects in meconium and fecal samples

**Figure 5:**
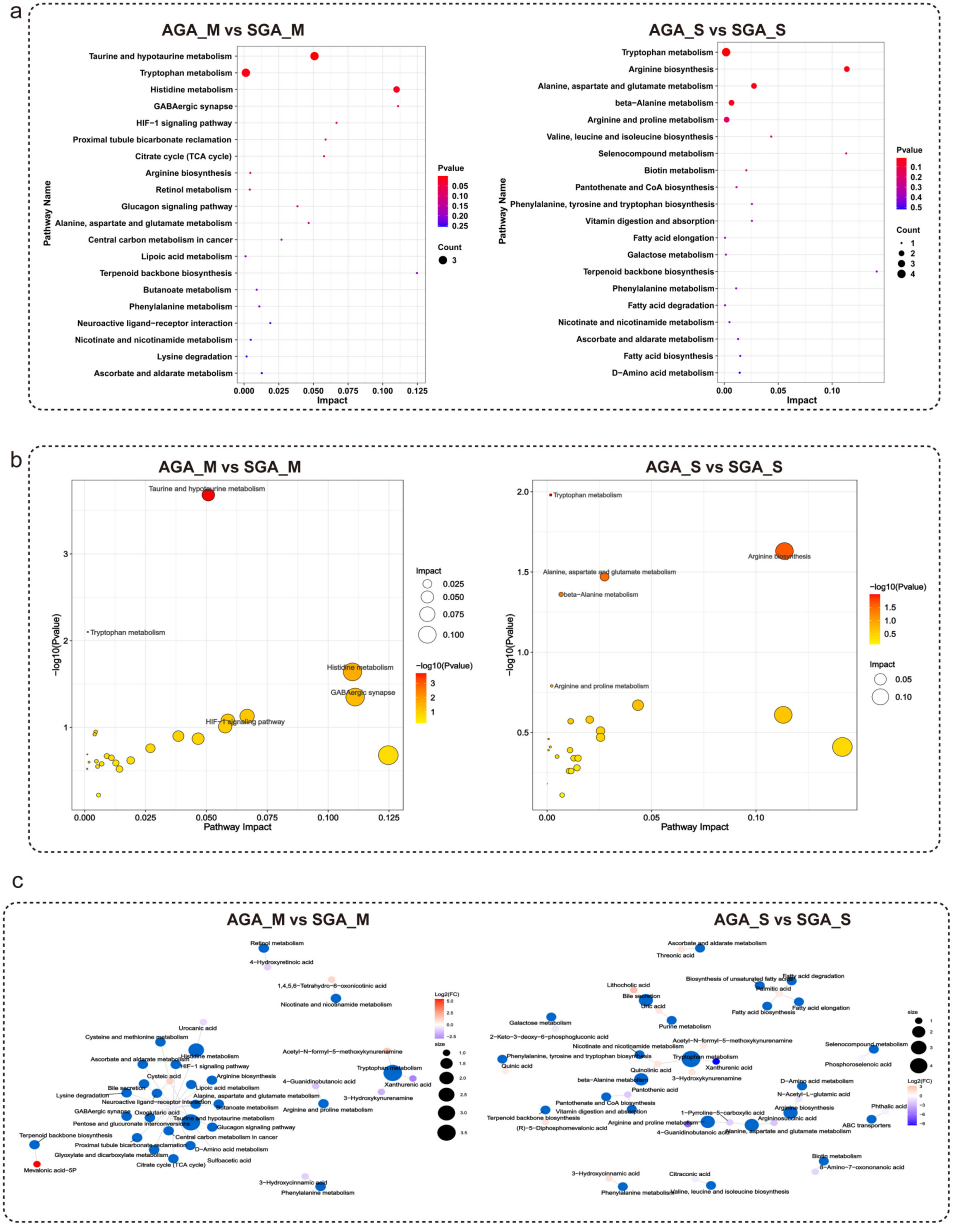
Metabolic pathway enrichment and functional analysis reveal altered metabolic networks in SGA and AGA subjects. **(a)** KEGG pathway enrichment of differential metabolites between AGA and SGA groups in maternal **(left)** and newborn subjects **(right)**; **(b)** Bubble plots for key enriched metabolic pathways in AGA and SGA groups; **(c)** Functional enrichment maps based on differentially abundant metabolites.

To gain mechanistic insight into the metabolic disturbances associated with SGA maternal and neonatal subjects, pathway enrichment and topology analyses were performed using differential metabolites identified between SGA and AGA groups. In maternal participants (AGA_M vs. SGA_M), significant enrichment was observed in taurine and hypotaurine metabolism, tryptophan metabolism, and histidine metabolism pathways (Figure 5a, left), with high pathway impact and strong statistical significance (FDR-adjusted *p* < 0.05). Additional alterations were noted in central carbon metabolism, citrate cycle (TCA), and arginine biosynthesis, suggesting disrupted energy and amino acid metabolism in SGA neonates. In neonatal subjects (AGA_S vs. SGA_S), tryptophan metabolism again emerged as a key altered pathway, alongside beta-alanine metabolism, arginine and proline metabolism, and pathways related to branched-chain amino acid biosynthesis (valine, leucine, and isoleucine) (Figure 5a, right). These findings suggest systemic perturbations in tryptophan metabolism in the SGA group.

Bubble plot visualization further highlighted the significance and metabolic relevance of these pathways. In maternal subjects (Figure 5b, left), taurine/hypotaurine and tryptophan metabolism were characterized by both high impact and low *p*-value, while in neonatal participants (Figure 5b, right), tryptophan metabolism, along with alanine, aspartate, glutamate metabolism, and arginine and proline metabolism were prominently enriched.

Functional enrichment analysis of key differential metabolites (Figure 5c) reinforced these findings. In maternal subjects, metabolites involved in kynurenine pathway, pyrimidine metabolism, and oxidative stress response were enriched in the SGA group, including kynurenic acid, uracil, and 4-hydroxyphenylacetic acid. In neonatal participants, dysregulation was evident in galactose metabolism, bile acid biosynthesis, and linoleic acid metabolism. Notably, common enrichment across matrices in tryptophan and taurine-related pathways points to consistent metabolic stress and adaptive shifts in SGA groups. Together, these data reveal that SGA neonates exhibit broad metabolic pathway alterations spanning amino acid metabolism, energy production, and redox balance, with both shared and matrix-specific signatures across maternal and neonatal samples.

### Distinct microbiota-metabolite correlation networks in AGA and SGA dyads reveal potential microbial contributions to growth programming

**Figure 6:**
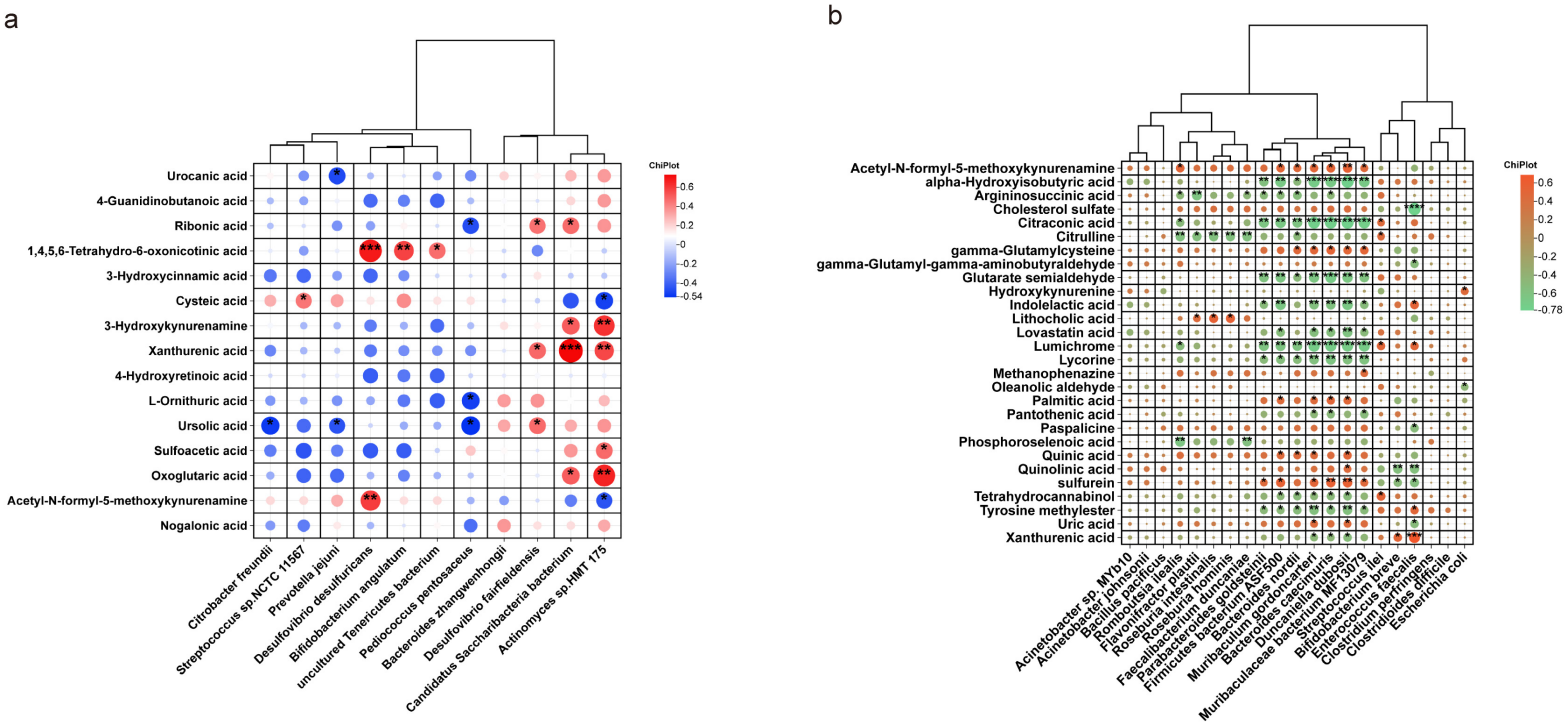
Spearman correlation analysis between differential metabolites and microbial species in neonates and their mother. **(a)** Correlation matrix in the AGA_M and SGA_M groups; **(b)** Correlation matrix in the AGA_S and SGA_S groups. The *p*-value was calculated using the two-tailed unpaired Students' *t*-test. *p < 0.05. Significant *p*-values were corrected using the Benjamini–Hochberg method. **p < 0.01, ***p < 0.001

To investigate the potential interactions between gut microbiota and metabolic profiles, we performed Spearman correlation analyses between differential metabolites and microbial taxa in both neonates and maternal samples. In maternal subjects (Figure 6a), strong correlations were observed between specific microbial species and amino acid or bile acid-related metabolites. Notably, 1,4,5,6-Tetrahydro-6-oxonicotinic acid showed a significantly positive correlation with *Desulfovibrio desulfuricans, Bifidobacterium angulatum*, and *uncultured Tenericutes bacterium* while urocanic acid was inversely correlated with *Prevotella_jejuni*. Several other host-derived metabolites (e.g., cystic acid, 3-hydroxykynurenamine) displayed significant associations with *Candidatus Saccharibacteria bacterium* and *Actinomyces sp.HMT 175*, suggesting that microbial activity may contribute to the modulation of tryptophan and histidine metabolism in AGA and SGA groups. In neonatal participants (Figure 6b), broader metabolic-microbiota associations were identified. Bacteroidota such as *Bacteroides caecimuris, Duncaniella dubossi, Muribaculum gordoncarteri* exhibited strong positive correlations with acetyl-N-formyl-5-methoxykynurenamine, gamma-glutamylcysteine, and palmitic acid, while negative correlations with alpha-hydroxyisobutyric acid, citraconic acid, indole-3-lactic acid, and xanthurenic acid. Firmicutes such as *Enterococcus faecalis* was positively correlated with indole-3-lactic acid and xanthurenic acid, while negatively correlated with uric acid and quinolinic acid. Proteobacteria such as *Escherichia coli* showed consistent positive relationships with hydroxykynurenine. These results reveal distinct microbiota-metabolite correlation networks in neonates and their mother, supporting the hypothesis that altered maternal and infant gut microbiomes may shape metabolic phenotypes related to intrauterine growth.

### Altered SCFA profiles reflect microbial functional shifts in mother and infants

**Figure 7:**
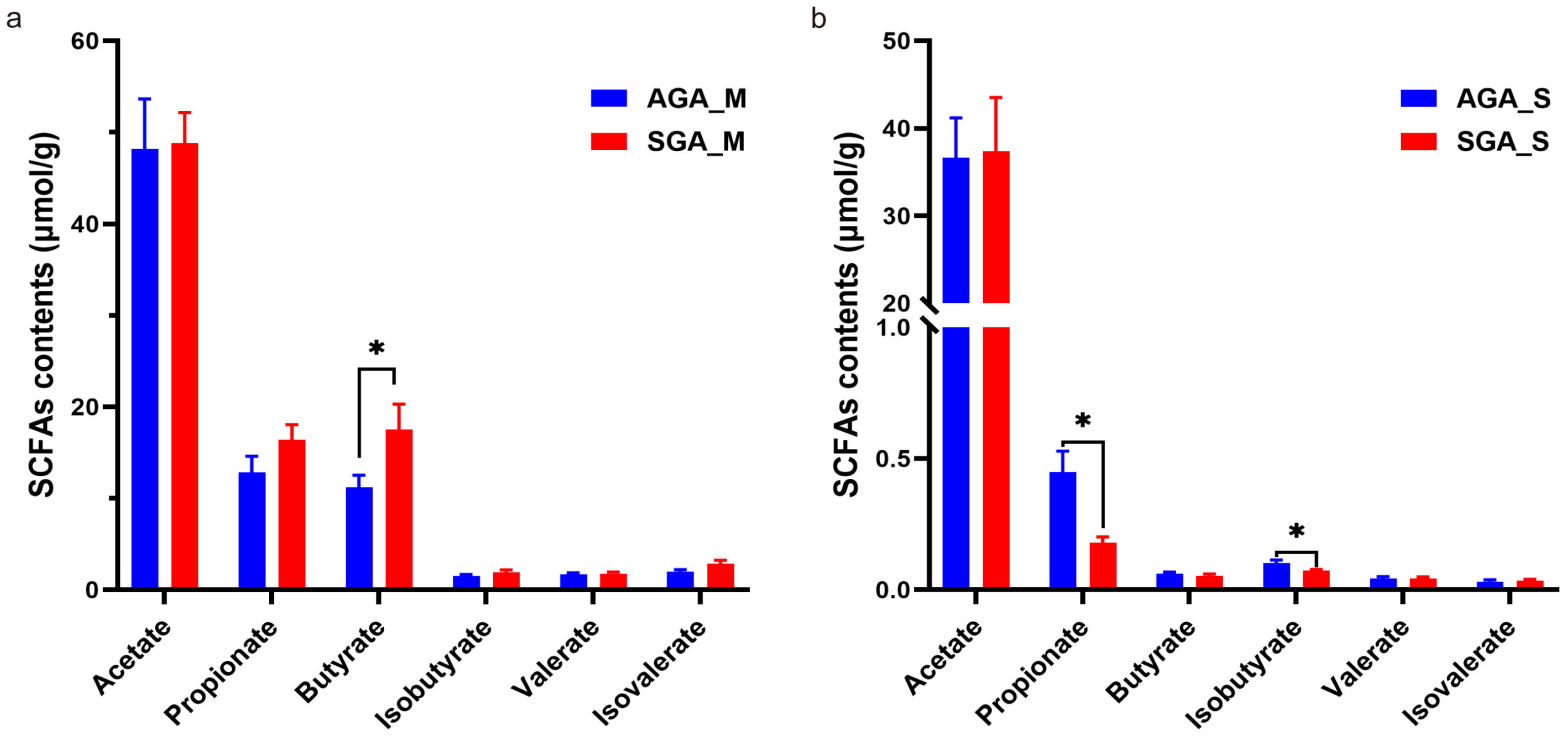
Altered SCFA profiles in maternal and infant fecal samples between AGA and SGA groups. **(a)** Comparison of SCFA concentrations in maternal feces between AGA_M and SGA_M groups; **(b)** SCFA concentrations in infant feces (AGA_S vs. SGA_S). Data are presented as mean ± SD. The *p*-value was calculated using the two-tailed unpaired Students' *t*-test. *p < 0.05. The *p*-value interaction between groups was corrected by Bonferroni-correction.

To explore functional shifts in microbial fermentation, we quantified fecal SCFAs in both mother and infants. Quantification of fecal SCFAs revealed significantly elevated levels of butyrate in SGA_M group compared to AGA_M group (*p* < 0.05, Figure 7a), whereas in infants, SGA_S samples exhibited significantly lower levels of propionate and isobutyrate relative to AGA_S counterparts (*p* < 0.05, Figure 7b), suggesting that the observed differences in SCFA profiles may represent an early metabolic signature in SGA infants.

## Discussion

This study provides a multi-omics characterization of the metagenome and metabolome in SGA neonates and their mother, revealing distinct microbial, metabolic, and functional signatures compared to AGA counterparts. Our findings support the notion that SGA is associated with early-life alterations in the maternal-infant gut axis, potentially influencing neonatal development and metabolic programming.

Although alpha diversity did not differ significantly in Shannon and Simpson indices, a reduction in Chao1 richness in SGA neonates suggests a contraction in microbial species richness, consistent with previous observations linking low birth weight to reduced microbial complexity early in life (Chang et al., 2022; Chen et al., 2022). Notably, beta diversity analyses via PCoA failed to reveal significant compositional divergence, while PLS-DA demonstrated clear separability, indicating subtle but biologically relevant shifts in microbial taxa. Taxonomic profiling revealed an increased abundance of Tenericutes in SGA mother and reduced Synergistetes in SGA neonates, echoing earlier reports implicating shifts in low-abundance phyla in adverse perinatal outcomes (Li et al., 2019). Tenericutes are important constituents of the gut microbiota, whose abundance and diversity may be modulated by hormonal and immune changes during pregnancy. Alterations in Tenericutes have been associated with various pregnancy complications, including preterm birth and fetal growth abnormalities (Saraf et al., 2022).

At the species level, SGA infants exhibited enrichment in opportunistic or conditionally pathogenic taxa such as *Escherichia coli* (facultative anaerobe) and *Enterococcus faecalis* (microaerophile). The gastrointestinal tract of neonates is colonized immediately by bacteria after birth. *Escherichia coli* is among the first bacterial species to colonize the neonatal gut within hours after birth, establishing a mutualistic relationship with the host (Villa et al., 2013). *Enterococcus faecalis* is also an early colonizer of the infant gut and contributes to immune modulation (Kanasugi et al., 1996; Satonaka et al., 1996). However, the role of *Enterococcus faecalis* in human health remains controversial. In immune-compromised individuals, *Enterococcus faecalis* can act as an opportunistic pathogen (Pinholt et al., 2014). Whereas, strains isolated from healthy adults have been shown to exhibit probiotic properties (Nueno-Palop and Narbad, 2011). During early colonization, facultative anaerobes such as *Escherichia coli* consume residual oxygen in the intestinal lumen, thereby reducing the local redox potential and facilitating the transition to an anaerobic environment (Dominguez-Bello et al., 2011; Byndloss et al., 2018). Previous studies have demonstrated that SGA infants exhibit higher oxidative stress at birth, the severity of SGA was significantly correlated with oxidative stress levels in SGA neonates (Ashina et al., 2021). Reactive oxidative metabolites can influence the redox potential of the gut environment, thereby potentially favoring taxa with specific adaptive mechanisms. Studies have reported elevated levels of lactic acid accumulation in SGA infants at delivery and in the first few postnatal hours (Hawdon and Ward Platt, 1993). Lactic acid serves as energy source for the aerobic growth of *Enterococcus faecalis* (Pritchard and Wimpenny, 1978; Clarke and Knowles, 1980; London, 1968), which may partly explain its high abundance in the meconium of SGA infants. Notably, metagenomic sequencing revealed no substantial overlap between differentially abundant microbial species identified in SGA infants and those in their mother. This finding suggests a limited contribution of maternal gut microbiota to the initial microbial colonization of SGA neonates, potentially arguing against a dominant role of vertical microbial transmission in this context. In parallel with alterations in microbial taxonomic profiles, we observed significant changes in the abundance of specific CAZymes. The reduced abundance of key glycoside hydrolases (GH20, GH13_16) and glycosyltransferases (GT75, GT100, GT33) in SGA maternal subjects suggests diminished capacity for complex carbohydrate degradation and host-gut mutualism. The upregulation of GH5_9 and PL1 alongside the marked downregulation of mucin- and fucose-targeting hydrolases (GH95, GH110), glycosyltransferases (GT5, GT33), and carbohydrate-binding modules (CBM6) in SGA newborn subjects, suggests a microbiota-level metabolic shift from host-glycan-dependent colonization toward plant polysaccharide-based metabolism, potentially driven by impaired mucosal development and altered postnatal nutrient exposures. In breastfed neonates, HMOs can modulate the expression and activity of specific GH families, which in turn influences bacterial composition and HMO utilization pathways, indicating a dynamic HMO-GH-microbiota interplay (Samarra et al., 2025). This bidirectional HMO-GH-microbiota axis may help contextualize the compositional and functional differences observed in early life. Taken together, our findings suggest that impaired microbial maturation in SGA infants may reduce debranching CAZyme activity, limiting complex carbohydrate utilization and exacerbating growth restriction.

The vertical transfer of metabolome from mother to infant has been best characterized in human subjects (Lovrić et al., 2024). Tryptophan metabolism plays a critical role in regulating infant growth and development. Tryptophan is an essential aromatic amino acid primarily metabolized through the kynurenine pathway, serotonin/melatonin pathway, and indole pathway by gut microbiota. Recent studies have demonstrated that maternal-fetal tryptophan transfer remains intact in fetuses with SGA, but its conversion to quinolinic acid is impaired (Sano et al., 2016). In the present study, 3-hydroxykynurenine and xanthurenic acid were increased in SGA mother, while 3-hydroxykynurenine and quinolinic acid were decreased and xanthurenic acid was increased in SGA infants. Quinolinic acid is a key downstream metabolite of the kynurenine pathway and responsible for nicotinamide adenine dinucleotide (NAD) synthesis. Kynurenine 3-monooxygenase, an essential enzyme of the kynurenine pathway, which converts kynurenine into 3-hydroxykynurenine (Badawy, 2017). 3-Hydroxykynurenine has been shown to possess anti-inflammatory and antioxidant properties by modulating STAT1 and NF-κB signaling (Clement et al., 2021). 3-Hydroxykynurenine is degraded by kynureninase into 3-hydroxyanthranilic acid and is further degraded by kynurenine aminotransferase into xanthurenic acid. Xanthurenic acid was found to activate type-2 and−3 metabotropic glutamate receptors (mGlu2 and−3 receptors) (Fazio et al., 2017). Previous study has identified a protein in *Escherichia coli* with kynurenine aminotransferase activity (Han et al., 2001). This may explain the elevated levels of xanthurenic acid in the meconium of SGA infants. Acetyl-N-formyl-5-methoxykynurenamine, a metabolite of melatonin, was known to be produced or modified by the gut microbiome, and has potent antioxidant and anti-inflammatory abilities (Mayo et al., 2005; Tan et al., 2007). The level of acetyl-N-formyl-5-methoxykynurenamine was decreased in SGA mother and infants. Apart from kynurenine and melatonin metabolites, we also observed a significantly increased level of indole-3-lactic acid, another tryptophan catabolite, in SGA infants. Increased excretion of indole-3-lactic acid may reflect a shift in tryptophan metabolism toward microbial indole pathways. Previous studies have shown that *Enterococcus* and *Escherichia* can metabolize tryptophan to produce indole derivatives (include indole, indoleacetic acid, and indole-3-lactic acid) (Roager and Licht, 2018; Lee and Lee, 2010). Indole-3-lactic acid, a tryptophan-derived microbial metabolite, serves as a crucial mediator in host-microbiota crosstalk (Gao et al., 2018; Aragozzini et al., 1979; Narayanan and Rao, 1976). This increase may represent a compensatory response to impaired host kynurenine metabolism or an early microbial adaptation that supports gut immune homeostasis in the context of SGA infants. Collectively, these results indicate that SGA neonates may induce metabolic disorder by disturbing tryptophan catabolism.

In the mammalian gut, obligate anaerobe bacterium produce SCFAs such as butyrate, a key metabolite central to maintaining host-microbiota symbiosis (Jha et al., 2014). Butyrate levels increase significantly throughout pregnancy despite overall SCFA stability, potentially counteracting heightened late-gestation inflammation due to its well-established anti-inflammatory properties (Martín-Grau et al., 2022; Liu et al., 2018). Studies have shown that fecal levels of SCFAs (acetate, propionate, butyrate, isobutyrate, valerate, isovalerate, and caproate) are significantly reduced in SGA rats (An et al., 2021). In the present study, fecal SCFA profiles further corroborated microbial functional divergence, with increased butyrate levels in SGA mother and reduced propionate and isobutyrate in SGA neonates. While elevated butyrate in mother may represent a compensatory microbial adaptation to the metabolic demands of pregnancy under growth-restricted conditions, the diminished SCFA levels observed in infants may reflect delayed colonization, and differences in initial bacterial species. These findings indicate potential interruptions in the early vertical transmission of microbial functions related to SCFA production.

In summary, our results demonstrate that SGA is associated with altered gut microbiota colonization and metabolic disturbances in newborns. These findings lay the groundwork for future mechanistic studies and microbiome-targeted interventions aimed at improving health outcomes in growth-restricted infants. Several limitations warrant consideration. Despite the use of a priori power calculations, the limited sample size due to the rarity of SGA cases may still constrain our ability to detect small to moderate effect sizes, particularly given the high-dimensional nature of the omics data and the multiple comparisons performed. As a result, some biologically meaningful associations may have been overlooked. Future studies with larger cohorts or independent validation sets are needed to confirm and extend our findings. Longitudinal data are needed to evaluate the persistence and developmental consequences of these microbiota and metabolic alterations. Additionally, maternal diet, antibiotic exposure, and perinatal stress-though controlled for-may still exert residual confounding effects.

## Data Availability

The sequences of raw data generated in the present study have been deposited at the NCBI SRA database under the accession number PRJNA1277160. Untargeted metabolomics raw data and metadata have been uploaded to National Genomics Data Center under the accession number OMIX011963. Standard curve, precision, limit of quantitation, quality control frequency, and absolute concentration data for SCFAs have been provided as Supplementary Tables 1, 2.
